# Physical activity on prescription (PAP) from the general practitioner’s perspective – a qualitative study

**DOI:** 10.1186/1471-2296-14-128

**Published:** 2013-08-29

**Authors:** Gerthi Persson, Annika Brorsson, Eva Ekvall Hansson, Margareta Troein, Eva Lena Strandberg

**Affiliations:** 1 Blekinge Centre of Competence, SE-371 81, Karlskrona, Sweden; 2 Department of Clinical Sciences in Malmö/ Family Medicine, Lund University, Jan Waldenströms gata 35 SE-205 02, Malmö, Sweden

**Keywords:** Focus group, Physical activity, Prescription, Primary health care, Promotion

## Abstract

**Background:**

Physical activity on prescription (PAP) is a successful intervention for increasing physical activity among patients with a sedentary lifestyle. The method seems to be sparsely used by general practitioners (GPs) and there is limited information about GPs’ attitudes to counselling using PAP as a tool. The aim of the study was to explore and understand the meaning of prescribing physical activity from the general practitioner’s perspective.

**Methods:**

Three focus group interviews were conducted with a purposive sample of 15 Swedish GPs in the south of Sweden. Participants were invited to talk about their experience of using PAP. The interviews were transcribed verbatim, analysed using qualitative content analysis.

**Results:**

The analysis resulted in four categories: The tradition makes it hard to change attitude, Shared responsibility is necessary, PAP has low status and is regarded with distrust and Lack of procedures and clear guidelines. Traditionally GPs talk with patients about the importance of an increased level of physical activity but they do not prescribe physical activity as a treatment. Physician’s education focuses on the use of pharmaceuticals. The responsibility for patients’ physical activity level is shared with other health professionals, the patient and society. The GPs express reservations about prescribing physical activity. A heavy workload is a source of frustration. PAP is regarded with distrust and considered to be a task of less value and status. Using a prescription to emphasize an increased level is considered to be redundant and the GPs think it should be administered by someone else in the health care system. Scepticism about the result of the method was also expressed.

**Conclusions:**

There is uncertainty about using PAP as a treatment since physicians lack education in non-pharmaceutical methods. The GPs do not regard the written referral as a prioritized task and rather refer to other professionals in the health care system to prescribe PAP. GPs pointed out a need to create routines and arrangements for the method to gain credibility and become everyday practice among GPs.

## Background

Evidence shows that physical activity can be used to promote health and to prevent and treat over 30 physical and mental illnesses [1]. An increase in physical activity is one of the measures that is said to have the greatest positive effect on public health [1]. Physical activity has been identified as the most important health-related behaviour to change, and patients ask health care staff for support in making lifestyle changes [2]. The health care system is in a good position to work for an increase in physical activity among the population, partly because many individuals have contact with the health service each year, and partly because they trust it [1]. Primary health care also reaches the groups that are most sedentary and vulnerable in society, for example young adults, single people, and immigrants. Lifestyle advice from general practitioners (GPs) has been shown to have a positive effect on the health of the population [3].

Physical activity on prescription (PAP) is an individually adjusted written prescription of physical activity that all health care providers in Sweden recommend their employed physicians to use in order to prevent and treat illness [1]. The Swedish National Institute of Public Health estimates that 28 000 PAPs were prescribed in 2009 and the use continues to rise [4]. PAP means that authorized health care staff issues an individual written prescription for the intensity, duration, and type of activity that the patient should perform in order to minimize a sedentary lifestyle [5]. The method is based on several theory-based behavior change models, but is primarily inspired by the transtheoretical model and social cognitive theory. The models describe progress through stages of change such as contemplation, preparation, action and maintenance as well as self-efficacy [1]. The routines for prescription and the layout of the prescription itself have been developed to resemble prescriptions for medicines, as a way to enhance the significance of the prescription. In Scandinavia as well as in other countries variants for prescribing physical activity exist [6-9]. There is evidence that PAP is a cost-effective method for use in primary care [10,11]. Physicians’ attitudes and their ability to communicate with patients have a significant impact on patient compliance. However, physicians are the professional group with the least positive attitude to doing preventive work in health care [12,13]. A Danish study found that doctors have ethical misgivings about showing concern for their patients’ lifestyle [14]. A study from the USA has found that only 35 per cent of patients with unhealthy habits regularly receive advice from doctors [15]. When advice is given it is more effective if the doctor presents his recommendations about physical activity as a detailed prescription. The effect increases further if the doctor follows up the prescription [16]. Despite studies showing that PAP is an effective complement to or substitute for medication, it seems as if PAP is not used to its full potential [17,18]. Attempts have been made to stimulate the use of PAP, and it was found possible to increase the number of prescriptions by doctors when they collaborated with physiotherapists in prescribing physical activity [19]. The use of PAP from a GP perspective, however, does not appear to have been studied previously.

The aim of the study was to explore and understand the meaning of prescribing physical activity from the general practitioner’s perspective.

## Methods

Forty-three GPs from 16 health care centres with experience of PAP were purposively selected and invited by e-mail. The selection included male and female GPs of different ages, with a varying number of years in the profession, working in publicly financed health centres located in urban and rural areas. Private health care centres were excluded due to lack of routines for prescribing PAP. Fifteen GPs from three counties agreed to participate, forming three focus groups. Some participants knew each other and some had never met before; in the smallest group all the participants were acquainted. No economic incentive was given for participation. Twenty-eight GPs declined participation, with shortage of time stated as the most common reason. Information about the participants in the focus groups is shown in Table 1. The non participants represented both genders and all age groups from every health care centre.

**Table 1:** Number of focus groups, population and participating GPs

**Focus group I-III**	**Population**	**No. of GPs (men/women)**	**Experience from general practice**	
			**<10 years**	**>10 years**
I Small town/countryside	64.100	6 (3/3)	2	4
II City	305.000	4 (0/4)	0	4
III Town	83.100	5 (1/4)	2	3

Data collection was done via focus groups. Based on the discussions in the focus groups, we searched for shared thoughts, opinions, and a meaning that can increase our understanding of how GPs view the prescription of physical activity. Focus groups are a semi-structured interview form with 7–12 participants who have some experience of the topic [20]. This data collection method is well known and tested as a way to seek an understanding of how people with similar experiences feel and think about a specific issue [20]. A focus group conversation invites discussion through participation. According to Morgan, the conversation generates data that is rich in viewpoints since the lively collective interaction can provoke more spontaneous expressive emotional opinions than an individual interview [21].

Three focus groups were conducted with the aid of a semi-structured interview guide according to Kvale [22].The guide included open-ended questions allowing a fluid conversation regarding the topic. After an opening presentation each participant answered the question ‘On what level are you physically active?’ Then a voluntary participant was asked to share the experience of prescribing PAP. This started a free association of the theme. The focus groups were conducted in 2011 immediately after the end of the working day in a room that was familiar to the participants so that an inviting atmosphere could be created. One focus group was led by one of the authors, GEP. Two focus groups were conducted by two of the authors, GEP as a moderator and ELS as an assistant. The moderator led the discussion and the assistant kept field notes and ensured that everyone had the opportunity to speak. The conversations lasted 75–90 minutes and were transcribed verbatim by a secretary. GEP listened to the recordings and read through the texts to clarify any obscurities. The first author (GEP) and EEH are physiotherapists, ELS is a behaviour scientist with experience of qualitative research. AB and MT are both GPs with experience of qualitative research and analysis.

### Analysis

The material was analysed with the aid of qualitative content analysis [23]. To get a feeling of the totality, GEP and ELS read through the transcriptions and listened to the recordings several times separately. The text was analysed individually by the authors to ensure credibility. Meaning units were identified as a first step and were then condensed and coded as they were expressed by the participants and perceived by the authors independently of each other. On the basis of the codes, subcategories were used as an intermediate stage to develop categories. We sought a deeper understanding of the meaning of the statements, and we met twelve times to discuss the coding of the meaning units, the subcategories and the categories until consensus was reached.

Two of the authors (GEP and ELS) participated in all steps. The other authors read all the material, reflected, commented and confirmed that they contained data supporting the findings.

### Ethical considerations

Ethical approval was granted by the regional ethics board in Lund, registration number 2010/703. The aim of the study and the focus group methodology was presented in the information letter, and informed consent was obtained from the participants. All the GPs took part voluntarily after working hours and were informed of their right to end their participation at any time. The material was de-identified and coded to guarantee confidentiality.

## Results

The results are presented in four categories with two to three codes per category (Table 2).

**Table 2:** Meaning units, codes and categories derived from the analysis

**Meaning units**	**Code**	**Category**
“We are supposed to work preventively, it’s one of our major tasks, yet it’s so difficult.” (B22)	**Prevention is part of the task**	**The tradition makes it hard to change attitudes**
“We are brought up to learn that diseases are treated by medical measures, which means that drugs often come first. Even if you try to change your attitude, the old ways hang on.” (B22)	**Habitual behaviour**	
“We are schooled in a multitude of pills.” (A14)	**Pharmacological training**	
“Since we don’t have much time to sit and talk about physical activity, I tend to refer patients to physiotherapists.” (B21)	**Someone else’s task**	**Shared responsibility is necessary**
“Physical activity is hard. Not everybody wants to take that path. You have to have the patient with you in all treatment contexts.” (B24)	**Patient’s role and expectations**	
“Patients have said themselves in the last few months, ‘But can’t I have a prescription?’ It’s interesting that wishes are expressed to me but I wasn’t the one who mentioned it.” (B8)		
“The structure of society can be changed by building cycle paths.” (C34)	**Society’s attitude**	
“To get through as many patients as possible in as short a time as possible, that’s our role.” (C37)	**High workload**	**PAP has low status and is regarded with distrust**
“It’s easy to forget, quite simply, among all the pills.” (A15)	**Low priority**	
“I suppose we’re not so convinced that it’s the actual PAP prescription that makes a difference.” (A19)	**Scepticism about PAP**	
“I can find it a bit complicated as it has been done, five different mobile phone numbers to choose among.” (C12)	**Vague routines**	**Lack of procedures and clear guidelines**
“There’s no institution for prescriptions for physical activity corresponding to what there is for ordinary prescriptions.” (B14)	**Unclear Processes**	

### The tradition makes it hard to change attitude

The shared view of the participants was that physical activity is essential for people’s health. It is traditionally a part of a doctor’s everyday work to talk about the importance of being physically active with patients who display a risk of developing illness. The participants said that they brought up physical activity when talking to the majority of patients. Depending on the reason for the consultation, the patient received varying amounts of information about the importance of physical activity as a way to affect their health status. The participants said that physical activity took up a large part of the consultation. In their view it is the doctor’s responsibility to inform people about the importance of being physically active, but there is no tradition of prescribing physical activity. One doctor put it as follows:

“I always emphasize that it is important to take action with patients who show risk of or already have developed illness.”

There was a feeling of constantly having a focus on physical activity, or as one doctor put it:

“We talk about physical activity every day, every hour, back and forwards, for every condition.”

The participants said that the meeting with the patient is important and that being a GP means ensuring in the encounter that the patient understands the importance of physical activity. The importance of putting forward one’s personal opinion of the significance of physical activity was stressed as a way to motivate patients to be more physically active. The doctors also said that they set a good example by being physically active themselves. In addition, the doctors considered that preventive work takes high priority and that the identification of high-risk lifestyles is part of a doctor’s responsibility for making a diagnosis and encouraging a desirable change in lifestyle. On the other hand, the GPs thought that actually prescribing physical activity is not necessary; the doctor’s responsibility is to talk about the importance of physical activity for achieving a change of behaviour in the form of a higher level of physical activity.

The GPs’ opinion was that physical activity in certain contexts can be preferable to pharmacological treatment. This applies, for example, to hypertension and diabetes for secondary preventive purposes. Moreover, the participants thought that virtually all pathological states benefit from increased physical activity, but doctors have no tradition of telling patients how to go about this in practice. Although there is knowledge about the importance of physical activity, the doctors felt that it takes time to change a treatment strategy from being geared to prescribing drugs to replacing or supplementing this with PAP. It may feel like a challenge to wait before starting pharmacological treatment, which usually leads to a quick recovery compared to improvement as a result of increased physical activity, which takes longer to see. The GPs thought that it would take a change in professional role for doctors and for other staff if PAP is to be used to a greater extent. There is ample knowledge of the importance of physical activity for health, but PAP is rarely used by doctors. The health care system often conveys double standards according to one of the doctors, who observed:

“We talk about this (physical activity), but we write prescriptions (for drugs). We talk about this, but we refer people to surgery for overweight, we talk about this, but we treat blood pressure, we talk about this, but we prescribe sleeping pills. You can mention one area after the other where we have double standards.”

Regardless of the number of years in the profession, the participants agreed that medical training is geared to science and lacks teaching about non-pharmacological methods, which results in uncertainty about using PAP. An experienced doctor expressed:

“I basically think that we don’t have any training in this, we have just been taught about molecules and pills for five and a half years.”

Younger GPs were able to tell about many occasions during their studies when physical activity was mentioned as first-line treatment for several diagnoses. On the other hand, there was no training in how to prescribe and dose PAP.

Motivational interviews (MI) were brought up as a possible method for stimulating a change in behaviour. Training in MI is not a part of the basic education of a doctor. The participants thought that MI is an art form taking not only education but a great deal of practice to master. Moreover, the GPs thought that it takes time and requires skill to meet the patients where they are in order to achieve a change in behaviour.

### Shared responsibility is necessary

The responsibility for increasing the level of physical activity is shared by the care team, the patient, and society. The participants felt that they lacked time for a dialogue with the patient about the dose and intensity of physical activity but the GPs felt responsible for underlining the importance of physical activity to promote health and treat illness. One GP explained:

“We have a nurse who has motivational interviews or health conversations.”

The responsibility for motivating the patient to engage in more physical activity is shared by several professions in health care and the doctors agreed that teamwork is necessary. It was considered suitable to refer to nurses and physiotherapists for advice about the dose and intensity of physical activity. According to the participants, increased physical activity is a major lifestyle change and it requires efforts by several professions to motivate increased physical activity. Shared goals and outlooks in health care were considered necessary to achieve results.

Even when the doctor recommends treatment with physical activity, the patient sometimes asks for medicine. Not all patients are prepared to change their lifestyle. Patients’ different needs for intervention were discussed, and it is far from always sufficient to increase physical activity to regain health. The participants thought that patients themselves have a great responsibility for their health and changes in lifestyle. The doctors must be able to make demands of the patients, or as one GP put it:

“We should perhaps be more unambiguous and say no, you have responsibility for your health, the responsibility for your health is yours alone, it’s best for you to do this or that, to take responsibility for your health.”

The GPs thought that health care alone should not be responsible for promoting the citizens’ physical activity. Society’s attitude to medications must be changed so that it becomes generally accepted that drugs are not always necessary to get well. School has a great responsibility for making it possible for children to engage in physical activity, and society must stimulate an active physical life, for example, by building cycle paths and playgrounds and offering subsidized physical activities near residential areas. Everyone must take responsibility. One doctor said:

“The optimal thing really would be to have a society where people move.”

### PAP has low status and is regarded with distrust

The participants expressed frustration about the pressure of their work situation. The intense working tempo was considered to result in difficulties in finding time for motivational interviews and prescriptions of physical activity. Some expressed a sense of inadequacy when it came to influencing patients to increase their physical activity. The participants said they wanted to do more primary preventive work and felt frustrated that secondary prevention takes up the greater part of a doctor’s working day. One experienced doctor said:

“I can contribute what I as a person think is correlated to health, but then I can’t do much more. For the individual that you have in front of you in that encounter and with his or her problems, it feels as if you have very little to contribute. It feels as if we ought to come in much earlier for the problems that our patients have. If we had come in earlier we would have had more chance of making a difference. When we see the patient it is at the level of secondary prevention instead of primary prevention.”

It emerged from the conversations that PAP has low status and low priority as a treatment option. Pharmaceutical treatment is used in the first instance and enjoys good support from the medical establishment. One doctor pointed out that routines and working methods for the handling of drugs are so solidly established that it is easy to forget alternative treatments. Colleagues, nurses, and patients expect quick treatment results, which can mean that medication takes priority over treatment with physical activity. Moreover, the participants felt that physical activity is not medicine but something obvious that should not need to be prescribed:

“I have a lot to say about this (PAP) and I was a bit doubtful when it (physical activity) came on prescription, since I view this as self-evident.”

There is distrust about PAP, as some doctors thought that the method lacks credibility and significance for the patient. The method is an attempt at a simple solution to a complex lifestyle problem, or as one GP put it:

“We know that physical activity is good but I’m not sure that a slip of paper is enough.”

Another doctor said:

“We don’t prescribe PAP because we don’t believe in the slip (the prescription).”

Even though the participants were convinced that physical activity is an important factor in preventing and treating illness, many were doubtful that a prescription can make a difference. Others thought, in fact, that PAP appeared to have some magical quality for the patient, which the majority of the GPs said they could not understand. While the doctors said that there was an excessive belief in PAP, in their experience the credibility and significance of the method nevertheless increases for the patient and for the doctor if the prescription resembles a prescription for medicine. The appearance of the prescriptions for drugs has changed a few years ago, so the PAP no longer resembles a drug prescription. The change was perceived as a reduction in the significance of the method, or as one doctor put it:

“The power has gone out of the prescription now that it’s been changed to an ordinary paper.”

The doctors questioned the degree of compliance with the method and the equivalence of the outcome to pharmacological treatment. The opinion was that the expected effect of increased activity takes time and can therefore be difficult to compare with other treatments. There was uncertainty among the doctors as to which diseases and conditions to treat with physical activity and how to prescribe PAP. The actual prescribing of physical activity was deemed to be an unnecessary task for doctors. Some participants were sceptical about the existing evidence for PAP and doubts about the long-term effect.

“Is there evidence that the effect of physical activity persists?”

### Lack of procedures and clear guidelines

It was clear from the statements that the routines for PAP vary. The doctors expressed some frustration over vague prescription routines. They called for a coordination function where the patient could get assistance for the behaviour change needed to increase the level of physical activity.

There were no clear guidelines for keeping records of prescriptions of physical activity. The doctors wished for cooperation with other health care staff and feedback from contact persons outside health care who provide physical activity. One doctor said, with some exasperation:

“I don’t know who to refer to or how to act.”

## Discussion

### Summary of the results

Reasons such as attitudes, lack of training, distrust and organizational issues appeared to prevent GPs from prescribing PAP. Ambivalence was evident in the discussions. Physical activity was considered important for health and it was important for GPs to acknowledge the need to influence patients to increase their physical activity. Prescribing physical activity was a task that doctors did not feel comfortable performing. They thought that it is a natural task for doctors to talk about physical activity, but prescribing it is a job for someone else. It was felt that a written prescription for physical activity can be significant for the patient, but there was distrust about the potential of PAP to make a difference. Pharmacological treatment is traditionally the method used for lifestyle-related diseases, and the doctors did not think they were adequately trained or experienced in prescribing physical activity. The doctors wanted clear guidelines and processes for PAP. They said that there are physiotherapists and nurses who are more skilled to use the method. It is not just the health care staff that has a duty to promote health; society and patients themselves have a great responsibility.

### Discussion of the method

The aim of the study was to explore and understand the significance of prescription of physical activity from a GP perspective. The GPs were purposively selected to have experience of the topic discussed [24]. We conducted three focus groups to collect data, a number recommended in recently published studies [25,26]. Focus groups and the method for analysis have been shown in previous studies to be credible and appropriate for studying GPs’ experiences [25]. The selection was confined to southern Sweden and none of the focus group reached the minimum numbers of participants, a limitation and a weakness of the study. However the participants were given good opportunity to share experiences and insights of the topic [20]. A large number of GPs declined to participate with lack of time as a reason not to take part in the study. The views of the nonparticipants can be different from the GPs participating in the study. We tried, however, to achieve a representative composition as regards experience, age, gender, and rural/urban location and the result from all three focus groups was consistent. Women were over-represented in the groups, but this may reflect how female doctors show more interest in preventive work [12]. The result cannot be generalized but may be transferable to similar contexts.

From three counties in southern Sweden, 43 GPs were invited personally by e-mail, which may be a limitation of the study since it could mean that only doctors with a special interest in the issue agreed to participate. As a physiotherapist working with the prescription of physical activity, GEP has a pre-understanding that GPs find PAP of minor significance. The other authors in the multidisciplinary research team makes up for the pre-understanding that GEP represents.

### Discussion of the results

The participating GPs share basically the same view of PAP. As in other studies, the participants found it important for doctors to influence patients to engage in more physical activity [27,28]. The dialogue with the patient was highly valued, but PAP was not a task to which doctor’s assigned high priority. They thought that physical activity is obviously desirable for everyone and were therefore doubtful about the necessity to prescribe it.

The doctors talked about their own physical activity but felt that it can be difficult to practise what one preaches. This study has not investigated whether physically active doctors use PAP more than physically inactive doctors, but some studies indicate that personal physical activity can make a doctor more inclined to influence patients to increase their level of physical activity [29,30].

During the conversations it became clear that there was insufficient knowledge about how to use PAP. It may reflect how the profession of doctor is more medically orientated, confirming what has been suggested by many and namely, that education about non-pharmacological methods is inadequate. Other studies have shown similar results [31,32]. The doctors thought that motivational interviews are an art, and studies testify that doctors lack sufficient training in giving advice on lifestyle [33,34].

Health care is to a large extent organized on the basis of the development of medical competence [35]. PAP was developed when the Swedish National Institute of Public Health was commissioned by the Swedish government to make 2001 into Physical Activity Year, in consultation with authorities and organizations [36]. The non-medical origin of the method may be an explanation to why the prescription of physical activity encounters resistance from doctors. Earlier studies have shown that directives from authorities are not always well received. A sense of ownership and autonomy with regard to one’s professional role is an important motivational factor for the use of new methods [37].

The GPs thought it was their duty to talk about the importance of physical activity, but that prescribing it is a task for someone else. Earlier studies have shown that a whole team has the best long-term effect in achieving behavioural change in the patient, compared with intervention by just a doctor [38]. Doctors and nurses are usually associated with a health-promoting professional role, but other professions such as psychologists, counsellors, and physiotherapists have knowledge about attitudes to promote health and prevent illness.

In the health care system the competition between different professions can be perceived as hard [35]. There is sometimes a struggle about who should be permitted to prescribe medicines, but when it comes to PAP we find the opposite situation: it is a task that doctors would prefer not to perform. Doctors have reservations about using PAP because they give priority to other tasks. The method may be important for the patient, but doctors would rather have someone else in health care performing the task. Obstacles and difficulties in cooperation need to be identified from a GP perspective and from the point of view of other staff categories. Through increased cooperation between professions, the competence of different staff categories can be utilized and the use of PAP can increase [19].

This study with a qualitative approach aimed to shed light on GP’s perspective on how to use PAP. A greater understanding of the GP’s perspective on PAP could give opportunities to stimulate the implementation of the method in primary care.

It is a long term process to implement effective programs and new models such as PAP [39]. For more than a decade, research has pointed out obstacles that must be overcome to optimize advisory work in primary care, and the results indicate a number of organizational and individual obstacles that need to be overcome [27]. The Swedish National Board of Health and Welfare recently published national guidelines for preventive methods to support the use of PAP [40]. Our study indicates a lack of clear organizational guidelines at management level and on the level of everyday practice. Support for health-promotion work is of great importance for healthcare. Routines and processes must be made more explicit if PAP is to gain in credibility and become a natural and high-priority treatment option for doctors.

## Conclusion

Doctors have inadequate training in non-pharmacological methods, which means that there is uncertainty about prescribing physical activity. PAP is not a priority for GPs because other tasks are considered more important. It was deemed suitable to refer to nurses and physiotherapists for prescriptions of physical activity. The competences of different professions need to be utilized to achieve optimum teamwork in PAP, which would be in keeping with the inter-professional character of primary care. The GPs point out that the proper conditions have to be established in society and in the health service to increase the level of physical activity among patients and to support primary and secondary preventive work.

## Abbreviations

PAP: Physical activity on prescription; GP: General practitioner; WHO: World Health Organization.

## Competing interests

The authors declared that they have no competing interests.

## Authors’ contributions

GEP, ELS, EEH and MT conceived the design of the study. GEP and ELS carried out data collection, analysed data and drafted the manuscript. AB, EEH and MT performed a corroborative analysis and contributed to the development of the manuscript. The final manuscript was read and approved by all authors.

## Pre-publication history

The pre-publication history for this paper can be accessed here:

http://www.biomedcentral.com/1471-2296/14/128/prepub
